# Hybrid approaches for the detection of networks of critical residues involved in functional motions in protein families

**DOI:** 10.1186/1471-2105-16-S3-A8

**Published:** 2015-02-13

**Authors:** Dagoberto Armenta-Medina, Ernesto Perez-Rueda

**Affiliations:** 1 Departamento de Ingeniería Celular y Biocatálisis, Instituto de Biotecnología, UNAM Av. Universidad 2001, Cuernavaca, Morelos CP 62210, México; 2 Unidad Multidisciplinaria de Docencia e Investigación, Sisal Facultad de Ciencias, UNAM, Sisal, Yucatán, México

## Background

Currently there is great interest in identifying critical residues in proteins, to improve our understanding and allow for the engineering of protein families. Diverse approaches combine sequence information, structural data, dynamics analysis and functional description to determine the importance of amino acids with regards to protein function. In this work, we propose a hybrid approach for the identification of critical residues in proteins, combining the use of evolutionary information (co-evolution), cross-correlation of atomic fluctuations derived from Anisotropic Normal Mode Analysis simulations [1] (ANMA) and network analysis. Subsequently we have compared this method to existing approaches.

## Results

By combining the information of the covariance matrix derived from Statistical Coupling Analysis (SCA) [2] and the cross-correlation matrix of atomic fluctuations derived from ANMA, it was possible to identify a network of evolutionarily coupled residues involved in relevant motions in protein families. The outstanding sites revealed by our hybrid approach (ANMA.SCA) showed a high correspondence with experimental data, confirming the critical role of these sites in the functional mobility of proteins. In addition, our approach was found to be complementary to previous approaches. It maintained a good correspondence with approaches derived from extensive molecular dynamics, while being faster and less expensive in terms of computational resources [3].

## Conclusions

The hybrid approach ANMA.SCA opens a wide range of possibilities in the study of functional motion within protein families. By means of detecting networks of critical sites and their topology it is able to reveal the hidden aspects of protein dynamics.
